# Higher BCG‐induced trained immunity prevalence predicts protection from COVID‐19: Implications for ongoing BCG trials

**DOI:** 10.1002/ctd2.60

**Published:** 2022-06-05

**Authors:** Samer Singh, Dhiraj Kishore, Rakesh K. Singh, Chandramani Pathak, Kishu Ranjan

**Affiliations:** ^1^ Centre of Experimental Medicine & Surgery Institute of Medical Sciences Banaras Hindu University Varanasi Uttar Pradesh India; ^2^ Department of General Medicine Institute of Medical Sciences Banaras Hindu University Varanasi Uttar Pradesh India; ^3^ Department of Biochemistry Institute of Science Banaras Hindu University Varanasi Uttar Pradesh India; ^4^ Amity Institute of Biotechnology Amity University Gurgaon Haryana India; ^5^ Department of Pathology School of Medicine Yale University New Haven Connecticut USA

**Keywords:** Bacillus–Calmette–Guérin (BCG), COVID‐19, LTBI, SARS‐CoV‐2, trained immunity, tuberculin sensitivity test (TST)

## Abstract

Endeavors to identify potentially protective variables for COVID‐19 impact on certain populations have remained a priority. Multiple attempts have been made to attribute the reduced COVID‐19 impact on populations to their Bacillus–Calmette–Guérin (BCG) vaccination coverage ignoring the fact that the effect of childhood BCG vaccination wanes within 5 years while most of the COVID‐19 cases and deaths have occurred in aged with comorbidities. Since the supposed protection being investigated could come from heterologous ‘trained immunity’ (TI) conferred by exposure to *Mycobacterium* spp. (i.e., environmental and BCG), it is argued that the estimates of the prevalence of TI in populations currently available as latent tuberculosis infection (LTBI) prevalence would be a better variable to evaluate such assertions. Indeed, when we analyze the European populations (24), and erstwhile East and West Germany populations completely disregarding their BCG vaccination coverage, the populations with higher TI prevalence consistently display reduced COVID‐19 impact as compared to their lower TI prevalence neighbors. The TI estimates of the populations not the BCG coverage *per se*, negatively correlated with pandemic phase‐matched COVID‐19 incidences (*r*(24): −0.79 to −0.57; *p*‐value < .004), mortality (*r*(24): −0.63 to −0.45; *p*‐value < .03), and interim case fatality rates (*i*‐CFR) data. To decisively arrive at dependable conclusions about the potential protective benefit gained from BCG vaccination in COVID‐19, the ongoing or planned randomized controlled trials should consciously consider including measures of TI as: (a) all individuals immunized do not respond equally, (b) small study groups from higher background TI could fail to indicate any protective effect.

## 1 BACKGROUND

There have been efforts to understand and explain the differential impact of COVID‐19 on populations in pursuance of identifying protective variables that could predict the impact or be applied for intervention. Escobar et al.^1^ and Berg et al.^2^ had endeavored to explain/model the differential effect on populations based on ‘trained immunity’ correlates of countries as per Bacillus–Calmette–Guérin (BCG) vaccination rates after meticulous correction or fitting of the data for supposed major confounders like age, population density, development status, BCG coverage/implementation using the infections and mortality data from an early stage of pandemic (till April 22, 2020). However, other studies^3^, ^4^ have failed to find support for the association previously observed between BCG vaccination policy or coverage and the impact of COVID‐19 on populations when using updated data set. More recently, a study published by the Citizen science initiative of COVID‐BCG Collaborative Working Group in *Transboundary Emerging Diseases* in April 2021 goes on to indicate BCG childhood vaccination as a risk factor for COVID‐19.^5^ These conflicting assertions stem from fundamentally misplaced presumptions that BCG vaccination in childhood would provide lifelong protective or adverse effects completely disregarding the longevity of BCG vaccination conferred immunological correlates that seldomly last >5 years in the absence of revaccination, rechallenge, or exposure to environmental *Mycobacterial* spp.^6^, ^7^, ^8^

The extrapolation of associative observations made previously^1^, ^2^ linking BCG vaccination coverage to reduced COVID‐19 impact on populations was expected to disappear^3^, ^4^, ^8^ as the populations compared were at different phases of the wave‐of‐infections (*WoI*) so inherently inappropriate set for any correlative comparative analysis to indicate presumptive cause and effect association. Additionally, as the supposed ‘trained immunity’ conferred by childhood BCG vaccination usually wanes in <5 years,^6^, ^8^ the BCG vaccination or coverage during childhood should not have any logical bearing on the COVID‐19 outcomes in the most‐impacted adult elderly population. Hence, the premise of protective ‘trained immunity’ from BCG vaccination given in childhood or to children in a population is not supposed to decrease the severity of infection or supposedly provide any protection in currently aged as the BCG conferred ‘trained immunity’ correlates would have waned away long ago.^6^, ^7^, ^8^ The use of early‐stage pandemic data^1^, ^2^ when the populations were not evenly exposed along with displayed associations’ inherent disconnect with the mechanism proposed behind the observed protective correlation would make such assertions untenable. It was also highlighted by a study from Israel that found no significant association of the COVID‐19 incidence among individuals, with regard to their childhood BCG vaccination status (vaccinated *versus* unvaccinated).^9^ The same may apply to studies that are using disparate data sets from a later stage of the COVID‐19 pandemic and conclude the vaccinated countries to be more protected while overlooking the presence of countries with minimal COVID‐19 effect in no BCG vaccination policy countries as well.^10^ Similarly, the studies trying to correlate childhood BCG vaccination to higher COVID‐19 incidence or as a risk factor are also potentially indefensible due to gross overlooking of the basic facts about the longevity of trained immunity conferred from BCG vaccination and associated cross‐reaction.^5^ The conclusions drawn by the recent report of the Citizen science initiative of COVID‐BCG Collaborative Working Group may call for more conservativism and greater scrutiny due to the comparison of groups that have disproportionate representation of individuals from disparate underlying *Mycobacterium spp*. conferred background trained immunity as suggested by us previously.^6^

We reason, the dependability on the correlative associations as well as conclusions presented in previous studies^1^, ^2^, ^3^, ^4^, ^5^ would have tremendously improved on considerations: (a) direct measure of prevailing supposed protective ‘trained immunity’ correlate (TIC) as a result of populations exposure to *Mycobacterium* spp. or BCG vaccination,^6^, ^7^, ^8^ that is, Tuberculin positivity [TIC of BCG given at birth wanes within <5 years,^7^ so chances of supposed heterologous protection^5^, ^10^, ^11^ of elderly from childhood vaccination are remote]; (b) analysis of countries at a similar stage of the pandemic; (c) underlying confounders including potential contributory variables (e.g., Vitamin D, Zinc)^12^, ^13^, ^14^; (d) the correlations observed, at any time, to be the total sum of the effects from protective variable and preventive or curative measures in place (e.g., social distancing norms and adherence, medical infrastructure/support) and trained immunity being acquired as a result of natural infection or vaccination.

The European populations with quite dissimilar BCG coverage (including no vaccination)^15^ that have had almost simultaneous *WoI* during the pandemic but experienced differential COVID‐19 impact^16^ offer an excellent opportunity to evaluate the alternative hypothesis that if BCGs could be of any protective benefit the ‘trained immunity from *Mycobacterium* spp. exposure (BCG or environmental NOT necessarily the childhood vaccination coverage *per se*) would display protective covariation (negatively covary) with COVID‐19 incidence and mortality among socially similar countries as was suggested by us previously.^6^ It would be theoretically better equipped to predict the outcome or potentially flattened curve if any such association exists, that may have a cause and effect relationship. The current analysis of TIC and COVID‐19 data from 24 socially similar European countries, completely disregarding their vaccination coverage or policy, support a potential protective role for the prevalent TIC of populations on COVID‐19 incidence and mortality.

## 2 MATERIAL AND METHODS

The COVID‐19 incidence and mortality data for the European countries (Table 1) for first *WoI* (March to Aug 2020) was obtained from Worldometer and that for August 2021 and February 2022 from https://ourworldindata.org/covid‐cases [compiled from JHU CSSE COVID‐19 Data; Accessed on 28 March 2022] and that of East and West Germany states from https://www.citypopulation.de/en/germany/covid/ [Accessed on 10 October 2020] and previously published estimates.^6^, ^8^ The latent tuberculosis infection (LTBI)^17^ prevalence estimates for populations (i.e., ‘TIC’) were from Institute for Health Metrics and Evaluation.^18^ All statistical estimations and correlation analysis of the COVID‐19 incidence and mortality with TIC or LTBI prevalence of populations (average, standard deviation (STDEV), standard error, *F*‐value, correlation/Pearson coefficient (*r/R*), regression, etc.) were performed using Microsoft Excel 2019. The *p*‐values <0.05 were considered significant unless explicitly stated otherwise. The methodology employed has been essentially the same as described previously.^6^, ^12^, ^13^

**TABLE 1:** COVID‐19 data (cases per million [CpM] and deaths per million [DpM]) of European countries

**Countries**	**CpM 12 Mar 2020**	**DpM 12 Mar 2020**	**CpM 26 Mar 2020**	**DpM 26 Mar 2020**	**CpM 12 Apr 2020**	**DpM 12 Apr 2020**	**CpM 26 Apr 2020**	**DpM 26 Apr 2020**	**CpM 12 May 2020**	**DpM 12 May 2020**	**CpM 26 May 2020**	**DpM 26 May 2020**	**CpM 12 Jun 2020**	**DpM 12 Jun 2020**	**CpM 26 Jun 2020**	**DpM 26 Jun 2020**	**CpM 12 Jul 2020**	**DpM 12 Jul 2020**	**CpM 26 Jul 2020**	**DpM 26 Jul 2020**	**CpM 12 Aug 2020**	**DpM 12 Aug 2020**	**CpM 26 Aug 2020**	**DpM 26 Aug 2020**	**CpM 31 Aug 2021**	**DpM 31 Aug 2021**	**CpM 1 Feb 2022**	**DpM 1 Feb 2022**	**Population**	**LTBI 2017 (%)**
**Spain**	665.43	1.84	3118.24	93.35	4667.48	368.05	5243.71	495.96	5493.08	574.36	5622.58	595.47	5738.28	604.71	5819.68	606.06	6019.13	607.49	6584.73	608.11	8063.46	611.21	9581.54	619.60	103862.30	1804.25	214762.66	2003.05	46757733	6.06
**Iceland**	342.51	0.00	2347.79	5.85	4979.54	23.42	5245.93	29.27	5272.28	29.27	5281.06	29.27	5289.84	29.27	5327.90	29.27	5365.96	29.27	5406.94	29.27	5772.87	29.27	6094.88	29.27	29254.97	89.48	187577.28	124.73	341598	7.67
**Ireland**	14.15	0.20	367.73	3.84	1950.46	67.52	3892.64	218.94	4697.25	300.01	4999.08	325.68	5103.19	343.88	5136.34	348.93	5181.02	352.98	5232.17	356.21	5425.64	358.64	5733.94	359.24	70731.24	1021.89	238257.85	1231.41	4946514	8.14
Finland*	19.67	0.00	172.86	0.90	536.62	10.10	825.68	34.28	1083.16	49.62	1195.93	56.30	1276.23	58.64	1297.52	59.18	1315.92	59.18	1333.97	59.36	1378.89	60.09	1443.85	60.45	23065.37	190.51	88875.62	358.66	5542120	8.28
Netherlands*	35.82	0.29	433.52	25.32	1492.73	159.68	2207.86	261.07	2507.67	321.45	2659.00	341.64	2827.19	353.13	2917.27	356.05	2976.60	357.91	3088.85	358.20	3536.95	359.43	3973.74	362.58	113410.024	1051.70	268710.23	1244.51	17141028	8.29
Switzerland*	100.18	0.81	1363.11	22.16	2933.15	127.64	3353.93	185.81	3506.16	215.47	3550.13	221.01	3584.98	223.66	3633.80	226.43	3795.03	227.13	3971.49	228.17	4289.68	229.78	4690.84	231.17	89457.47	1250.53	258538.19	1459.81	8664751	8.42
Norway*	147.38	0.18	621.22	2.58	1202.10	23.58	1386.69	37.03	1502.76	42.00	1544.40	43.29	1588.06	44.58	1627.11	45.87	1654.56	46.43	1679.62	46.98	1802.32	47.16	1935.15	48.64	29305.69	148.93	147153.42	263.47	5428015	8.46
**Belgium**	34.40	0.34	537.60	41.30	2556.27	370.16	3977.84	627.10	4637.01	753.16	4953.97	797.05	5157.80	821.97	5268.77	829.30	5398.11	833.52	5667.21	836.63	6467.45	843.18	7108.87	851.72	101842.33	2181.85	273737.07	2500.19	11597764	8.75
Denmark*	116.30	0.00	323.87	7.07	1065.31	47.11	1479.60	72.82	1827.45	90.93	1971.87	97.14	2072.12	102.49	2187.04	104.22	2233.80	105.08	2318.69	105.77	2600.29	107.15	2853.42	107.50	59465.86	444.50	307559.28	648.51	5795502	8.81
**France**	44.04	0.93	446.50	25.97	1461.06	220.22	1907.82	349.69	2147.53	412.99	2229.12	436.54	2393.48	449.47	2495.31	455.66	2635.48	459.23	2795.92	462.50	3165.48	465.12	3883.60	467.77	100771.62	1698.01	290848.33	1948.61	65296963	8.86
Germany*	32.75	0.07	524.15	3.19	1525.21	36.05	1882.08	71.29	2065.81	92.31	2162.64	101.38	2233.77	105.73	2319.04	107.67	2385.26	108.96	2466.27	109.79	2634.58	110.66	2851.10	111.56	46994.44	1098.92	118928.37	1406.12	83827321	9.20
**UK**	7.89	0.13	155.54	12.92	1123.82	180.71	2038.02	352.76	3019.69	472.95	3536.57	532.41	3906.24	577.36	4125.05	592.38	4274.81	601.16	4406.99	605.08	4618.52	608.29	4840.00	610.29	99698.56	1944.52	256137.43	2301.89	67943420	9.55
Sweden*	76.27	0.10	296.36	13.26	1079.30	134.03	1865.89	253.13	2775.15	369.85	3523.17	438.21	4982.21	500.53	6450.65	536.53	7209.05	558.89	7504.02	568.68	7961.82	573.53	8291.71	575.31	110905.06	1446.04	214948.41	1570.94	10109375	10.07
Portugal*	7.65	0.00	347.73	5.89	1627.29	49.45	2324.22	88.60	2738.77	114.11	3042.34	131.67	3549.91	147.67	4009.69	152.57	4563.66	162.88	4921.99	168.47	5223.11	173.08	5521.49	177.30	102078.57	1745.00	264625.33	1963.82	10191814	10.33
**Czechia**	10.92	0.00	189.32	0.84	561.32	12.88	693.23	20.54	769.50	26.42	846.98	29.59	929.87	30.71	1032.56	32.39	1231.77	32.86	1432.48	34.63	1780.68	36.50	2142.51	39.02	156574.27	2834.99	283749.26	3472.69	10712210	11.41
**Estonia**	20.35	0.00	405.52	0.75	986.67	18.84	1238.43	36.93	1316.07	45.98	1382.40	47.49	1484.91	47.49	1496.97	47.49	1518.08	47.49	1533.15	47.49	1638.68	47.49	1741.94	48.24	107121.41	974.96	259631.09	1537.14	1326680	12.03
**Turkey**	0.01	0.00	42.96	0.89	674.17	14.18	1303.58	33.20	1674.60	46.09	1879.22	52.05	2074.01	56.56	2302.37	59.95	2521.14	63.48	2676.28	66.44	2892.80	69.73	3107.22	73.19	75119.07	666.84	137842.26	1030.26	84482851	12.46
**Slovakia**	3.85	0.00	41.39	0.00	135.90	0.37	252.56	3.30	268.31	4.94	277.10	5.13	282.41	5.13	300.91	5.13	348.16	5.13	399.08	5.13	492.67	5.68	647.61	6.04	143095.68	2302.69	292332.92	3275.67	5460073	12.70
Italy*	250.17	16.87	1333.08	136.33	2586.60	330.19	3269.98	441.89	3659.54	512.71	3814.12	546.69	3909.39	567.70	3970.11	576.73	4021.39	580.80	4071.91	583.34	4164.18	585.29	4343.26	586.59	75205.92	2140.57	184145.90	2433.84	60447245	12.87
Poland*	0.819	0	27.77	0.37	167.94	5.49	297.86	13.84	431.37	21.42	571.54	26.60	745.13	32.10	875.08	37.30	991.39	41.43	1126.18	43.97	1399.36	48.12	1666.54	52.24	76425.91	1993.41	130308.49	2789.48	37850425	12.88
Hungary*	1.66	0.00	27.03	1.04	146.02	10.25	258.90	28.17	343.09	44.01	390.52	51.68	419.73	57.48	427.39	59.86	438.47	61.62	459.28	61.72	493.77	62.65	547.62	63.59	84318.39	3119.94	162217.22	4304.58	9656316	13.03
Lithuania*	1.10	0.00	100.65	1.47	386.81	8.449	523.82	15.061	545.50	18.36	600.59	23.14	643.57	27.18	663.41	28.65	685.09	29.02	735.05	29.39	838.63	29.75	989.61	31.22	111093.06	1695.63	256029.86	2936.21	2725537	13.36
Bulgaria*	1.007	0.144	34.83	0.43	95.13	4.03	179.465	7.915	286.40	13.384	350.15	18.709	444.13	24.18	634.39	30.367	1032.61	37.71	1484.078	48.64	1974.83	67.79	2243.52	82.32	66081.60	2739.88	139648.57	4843.65	6953085	14.38
Ukraine*	0.00	0.00	2.584	0.09	57.42	1.669	185.783	4.596	357.80	9.329	485.78	14.245	664.70	19.53	914.81	24.398	1208.29	31.37	1461.78	36.36	1933.24	45.05	2517.17	53.00	54859.41	1311.44	98629.64	2463.63	43757273	15.95
**Average (for countries < 10% LTBI)**	130.04	0..40	867.68	20.37	2124.48	136.19	2786.82	228.00	3146.65	279.55	3308.86	298.10	3430.93	309.58	3512.90	313.42	3602.97	315.70	3746.07	317.17	4146.34	319.16	4582.58	321.65	72321.66	1077.09	220923.81	1290.91		8.37
**STDEV**	192.73	0.55	938.59	26.17	1420.07	128.68	1509.03	196.92	1539.49	236.51	1572.01	251.32	1583.06	260.70	1588.63	263.46	1619.76	265.23	1713.63	266.23	2009.51	267.91	2311.44	270.34	33503.91	735.13	70617.44	807.30 ‐		0.88
**Total Population of Countries with <10% LTBI**																													323282729	
**Average (for countries > 10% LTBI)**	31.15	1.43	237.43	13.45	708.71	49.15	1032.81	78.93	1263.84	102.22	1430.33	115.43	1677.50	126.35	1923.19	132.62	2147.42	137.72	2317.11	141.19	2566.15	145.39	2813.35	149.01	96906.53	1914.28	202009.08	2718.49		12.62
**STDEV**	72.22	4.87	372.47	38.89	765.70	96.01	1002.28	133.70	1184.71	163.84	1317.75	180.57	1600.43	194.35	1916.26	201.58	2091.14	205.55	2148.41	206.93	2215.65	206.71	2261.31	205.97	30842.19	756.12	68026.46	1137.86		1.61
**Total Population of Countries with >10% LTBI**																													283672884	

**1**. *Note*. *Countries are from Escobar et al. 2020^1^

**2. CpM**: Cases per million; **DpM**: Deaths per million; Feb: February; Mar: March; Apr: April; Jun: June; Jul: July; Aug: August. Years as indicated.

**3**. LTBI estimates from Global Burden of Disease Collaborative Network. Global Burden of Disease Study 2017 (GBD 2017) Results. Seattle, United States: Institute for Health Metrics and Evaluation (IHME)^18^

[https://gbd2017.healthdata.org/gbd‐search/; search term combination “Prevalence‐Latent Tuberculosis Infection‐Sex: Both‐Age: All Ages (Percent)” [Accessed on 06 April 2022].

**4. STDEV**: Standard deviation

## 3 RESULTS & DISCUSSION

**FIGURE 1:**
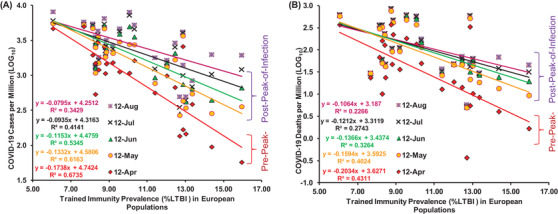
**The COVID‐19 cases (A) and deaths (B) in European countries with similar confounders and stages of pandemic consistently remained negatively and significantly correlated with trained immunity prevalence (est. %LTBI) starting from March 12 to August 26, 2020** . Refer **to** Figure 2 for correlation analysis for the period starting from 12 March to 26 August 2020, covering the duration up to April 22 of Escobar et al.^1^ and the August 1 reference point of Berg et al.^2^ and beyond up to 1st February 2022.

**FIGURE 2:**
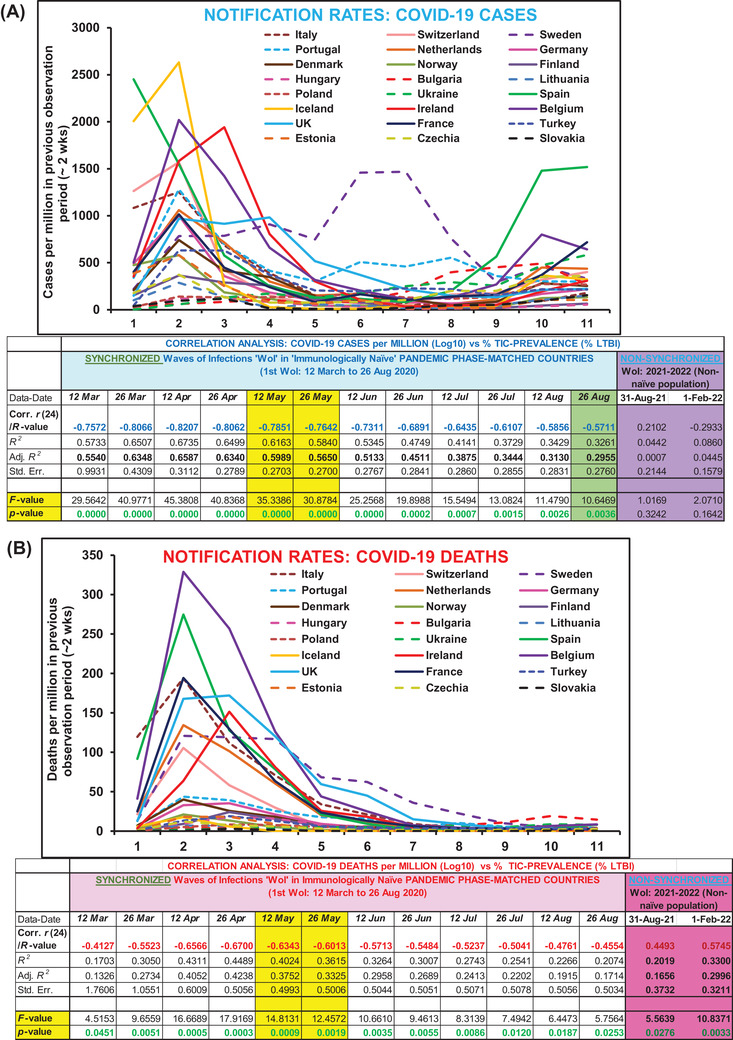
**The correlation between underlying prevailing trained immunity correlates (%LTBI) of European populations with COVID‐19 cases per million (A) and deaths per million (B) and its dependence on the phase of the pandemic**. The observed correlation (see bottom correlation analysis table) consistently remained negative for the period. The notification rates for countries with >10% LTBI is indicated by broken lines. The correlation remained high with the synchronicity of first peak of infections (see corresponding notification rates graph above for the data on date indicated in the table below) and been on decline since then partially resulting from the loss of synchronicity, populations response, acquired immunity, and understandably and importantly the changing reporting and management practices. ***Refer to Supporting information*** Figure S1 ***from European CDC that more accurately reflects the waves of infections or deaths from starting not explicitly observable in the figure presented here due to the coarse methodology employed***. The response of populations had been more stringent and uniform for first wave of infections. ***Note*. The highlighted 12** ^th^ **May *and*** 26^th^ **May values could** reflect the assumed total sum of actual maximum achievable correlation for potential ‘trained immunity’ along with current confounders and the stringent measures put in place by the countries to reduce the spread of COVID‐19—***Not*** necessarily due to only the prevailing trained immunity of the populations as a result of BCG coverage or implementation alone as assumed.^1^, ^3^ **Even if there is a cause and effect relationship**, **the expected protective covariation** (correlation) would expectedly further go down for the reasons mentioned above primarily due to increasingly heterogenous (loosened) response combined with changing trained immunity prevalence.

**FIGURE 3:**
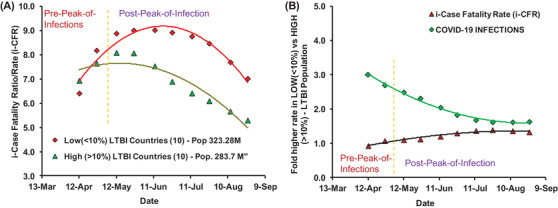
**The COVID‐19 cases, deaths, and CFR consistently remained lower in European populations with higher trained immunity (TI) correlate (>10% LTBI) post‐peak‐of‐infections than in countries with the lower TI‐correlate (<10% LTBI)**. The TI‐correlate indicated a significant consistently negative association with COVID‐19 infections (Pearson correlation *r*(24): −0.79 to −0.57, *p*‐value < .005) and mortality [*r*(24) = −0.63 to −0.45, *p*‐value < .05)] for the whole time period (12 March to 26 August 2020). Refer to Figure 1 in conjunction with Figure 2 for detailed correlation analysis and its variation with the wave of infections across 24 countries. Refer to Table 1 for updated COVID‐19 cases and deaths data for the 24 countries with supposedly similar confounders and at a similar stage of pandemic included in the study. (A) The i‐CFR [(deaths/cases)*100] for low LTBI countries had remained higher than that of high LTBI countries post infections peak. (B) Low LTBI countries have had relatively higher infections per million population (1.63‐fold on 26 August 2020) and consistently higher i‐CFR (∼30% on 26 August 2020).

Analysis of the updated COVID‐19 data (till 28 August 2020, Table 1,^16^ from 24 European countries with similar confounders (refer ^1^ and additional), stage of the pandemic and without any exclusions (applied in ^1^, ^4^) consistently displayed protective or negative correlation with the direct measure of desired heterologous TIC of populations (i.e., tuberculin positivity without active tuberculosis disease; referred by WHO as LTBI for the management purposes only due to slight chance of reinfection and reactivation in a small minority of individuals (WHO,^17^ IHME^18^). Higher LTBI prevalence populations consistently displayed lower COVID‐19 incidence and mortality per million (Table 1). The overall cases and mortality among European countries with similar confounders^1^, ^2^, ^6^ consistently remained negatively and significantly correlated with the prevalence of trained immunity correlate (%LTBI) for populations (Figure 1A & B). The correlative association displayed dependence on the phase of the pandemic (Figure 2A, B & Supporting information Figure S1). The countries with lower LTBI prevalence (<10%) reported higher incidence and fatalities during the study period (12 March 2020 to 26 August 2020) as compared to their higher LTBI prevalence (>10%) neighbors. Surprisingly, some outliers that had displayed lower deaths and infections despite lower TIC or higher infections and death despite relatively higher TIC prevalence are expectedly countries with higher Vitamin D and Zn sufficiency, respectively.^12^, ^13^ The correlation between TI estimate (%LTBI) and cases and mortality for COVID‐19 consistently remained negative post‐peak‐of‐infections (cases per million: *r*(24): −0.79 to −0.57; *p*‐value: < .004; mortality per million (*r*(24): −0.63 to −0.45; *p*‐value: <0.03). The i‐CFR among low (<10%) LTBI prevalence countries remained much higher than that among high LTBI prevalence countries (Figure 3A). With the progression of the COVID‐19 pandemic, the relative CFR between the groups of countries seem  to be steadily increasing while incidence rates had been falling (Figure 3B). The correlations observed shown here are supposed to decline further before the disappearance of COVID‐19 due to progressive loss of synchronicity of infections or pandemic phases (Figure 2A & B), lower prevalence of the protective variable (6.06–15.95%),^18^ the differential response of the study population, etc. not necessarily due to the supposed absence of correlation as proposed by Arlehamn et al.^3^ The apparent progressive closing of the gap in COVID‐19 impact on differential TIC prevailing populations (<10 and >10% LTBI) would be also being contributed by interventions or measures undertaken, for example, the introduction of vaccines and development of a gradual increase in herd immunity, changing COVID‐19 stringency measures (https://ourworldindata.org/grapher/covid‐stringency‐index accessed on March 28, 2022),^19^ confusions regarding fever management that controls pertinent immune responses (cell‐mediated and antiviral),^20^, ^21^ possible over prescription or self‐medication of supposed immune augmenting agents like Vitamin D^13^ and Zinc.^14^, ^22^, ^23^

**FIGURE 4:**
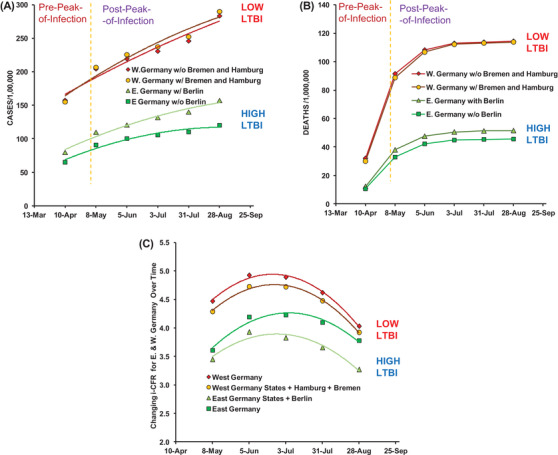
**East Germany (E. Germany) states with higher trained immunity correlate (%LTBI) as compared to West Germany (W. Germany) states (22.5** **vs. 9.2%) consistently reported lower COVID‐19 cases (A), Deaths (B), and i‐CFR (C) during the study period (10th April to 28th August)**. Refer to Table 2 for COVID‐19 cases and deaths and Table 3 for i‐CFR estimates. The E. Germany states consistently had 20–30% lower i‐CFR as compared to W. Germany states. The inclusion of Berlin in the East Germany region, and of Hamburg and Bremen in the West Germany region decreased the closing trend of the i‐CFR with the passage of time (compare covariation of red and green trend lines with orange and light green in [C], possibly indicative of more LTBI positives in Berlin than in Hamburg and Bremen. **In the future**, as the pandemic progresses, the gap between E. and W. Germany states is expected to close, partially resulting from a decrease in the vulnerable population and the concomitant increase in the population's overall ‘trained immunity’ as a result of infections and inoculations (asymptomatic or symptomatic; BCG or others, including SARS‐CoV‐2).

The East and the West Germany States that have been proposed in the early stage of the pandemic to be experiencing differential COVID‐19 impact^1^, ^3^ due to differential BCG coverage and policy provide a unique opportunity to test our assertion that actual trained immunity correlates (%LTBI) to be responsible for differential COVID‐19 impact. The estimated TIC (%LTBI prevalence) of East and the West Germany States are 22.5 and 9.2%, respectively.^7^ The East Germany States with higher TIC have experienced two‐fold cases while more than two‐fold fewer deaths from COVID‐19 per million populations during the study period (Table 2; data from https://www.citypopulation.de/en/germany/covid/). The inclusion or exclusion of city states did not change the supposed overall protective effect on populations. Similarly, the CFR for East and West Germany states remained significantly different for the whole period (Table 3). The COVID‐19 incidence and death rates remained significantly different between East and West Germany States (Figure 4A & B) both pre‐ and post‐peak‐of‐infections consistent with the potential protective role of TIC prevalence in populations. The CFR rates also remained consistently different during the study period (from 10th April to 28 August 2020; Figure 4C) without requiring any correction factors. However, the differential response gap seen for East and West Germany States is showing signs of closing as expected for populations slowly reaching toward stable equilibrium with underlying confounders (Figure 4C).

**TABLE 2:** COVID‐19 cases and deaths of erstwhile East and West Germany states. https://www.citypopulation.de/en/germany/covid/ [Accessed on 10 October 2020]

**EAST GERMANY (Region)**	**COVID‐19 Cases per 100k population**						**COVID‐19 Deaths per million population**					
**States∖Dates**	**10‐Apr**	**8‐May**	**5‐Jun**	**3‐Jul**	**31‐Jul**	**28‐Aug**	**10‐Apr**	**8‐May**	**5‐Jun**	**3‐Jul**	**31‐Jul**	**28‐Aug**
*Berlin: City State*	133.5	173.4	193.5	232.2	256.6	308.3	NA	44.42	54.5	58.32	60.23	60.77
Brandenburg	84.74	122.6	129.2	136.2	142	154.2	19.03	51.95	61.86	65.82	66.22	66.22
Mecklenburg‐Vorpommern [Mecklenburg ‐Western Pomerania]	38.31	45.46	48.13	50	55.09	62.93	6.84	11.81	12.44	12.44	12.44	12.44
Sachsen [Saxony]	94.33	121.2	130.8	134.1	136.4	147.2	15.72	45.19	52.06	55.01	55.26	55.26
Sachsen‐Anhalt [Saxony‐ Anhalt]	54.9	75.09	78.55	86.11	92.17	102.1	8.201	21.87	25.06	26.88	28.25	29.16
Thüringen [Thuringia]	73.55	122.3	145	153.5	158.6	169.7	11.25	52.5	79.22	84.84	85.31	85.31
**Average ( without Berlin)**	**65.27**	**91.01**	**100.62**	**105.93**	**110.57**	**120.48**	**10.50**	**32.84**	**42.20**	**44.79**	**45.32**	**45.54**
**Average (with Berlin)**	**79.89**	**110.01**	**120.86**	**132.02**	**140.14**	**157.41**	**12.21**	**37.96**	**47.52**	**50.55**	**51.29**	**51.53**

**WEST GERMANY (Region)**	**COVID‐19 Cases per 100k population**						**COVID‐19 Deaths per million population**					
*Bremen: City State*	72.52	164.1	225.6	246.9	262.8	292	19.08	46.98	64.59	77.8	80.74	82.21
*Hamburg: City State*	218.2	269	275.7	281.8	294.5	340.6	28.69	108.8	137	140.7	140.7	141.3
Baden‐Württemberg	235.7	301.5	315.5	323.2	337.6	378.5	55.31	136.5	160.2	165.7	165.7	166.4
Bayern [Bavaria]	264.7	340.3	361.5	371.7	389.7	437.2	54.93	161.1	189	197.9	199.3	199.8
Hessen [Hesse]	101.8	143.4	162.6	174.6	193.7	246.9	17.81	64.09	76.65	80.95	81.74	82.7
Niedersachsen [Lower Saxony]	102.8	136.7	156.7	171	182.1	209.8	21.39	61.67	75.56	79.19	80.56	81.44
Nordrhein‐Westfalen [North Rhine‐Westphalia]	150.1	195.8	215.7	245.8	275.7	327.8	27.97	77.84	90.1	93.94	95.34	96.56
Rheinland‐Pfalz [Rhineland‐ Palatinate]	121.1	154.3	165.6	173	184.9	219.7	14.66	46.9	56.18	57.4	58.14	58.38
Saarland	209.2	263.4	272.9	277.6	285.7	309.7	NA	141.9	168.2	176.3	176.3	176.3
Schleswig‐Holstein	76.76	101	107	110	119.9	139.6	NA	42.01	50.28	52.35	53.03	53.72
**Average (without Bremen & Hamburg)**	**157.77**	**204.55**	**219.69**	**230.86**	**246.16**	**283.65**	**32.01**	**91.5**	**108.27**	**112.97**	**113.76**	**114.41**
**Average (with Bremen & Hamburg)**	**155.29**	**206.95**	**225.88**	**237.56**	**252.66**	**290.18**	**29.98**	**88.78**	**106.78**	**112.22**	**113.16**	**113.88**

**TABLE 3:** I‐CFR rates in East and West Germany region or states

**EAST GERMANY (Region) i‐CFR RATES AT INDICATED DATES**					
**States∖Date**	**8‐May**	**5‐Jun**	**3‐Jul**	**31‐Jul**	**28‐Aug**
*Berlin: City State*	2.562	2.817	2.512	2.347	1.971
Brandenburg Mecklenburg‐Vorpommern	4.237	4.788	4.833	4.663	4.294
[Mecklenburg‐Western Pomerania]	2.598	2.585	2.488	2.258	1.977
Sachsen [Saxony]	3.729	3.980	4.102	4.051	3.754
Sachsen‐Anhalt [Saxony‐ Anhalt]	2.913	3.190	3.122	3.065	2.856
Thüringen [Thuringia]	4.293	5.463	5.527	5.379	5.027
**Average (w/o Berlin)**	3.609	4.194	4.229	4.098	3.780
**Average (with Berlin)**	3.450	3.932	3.829	3.659	3.274
**WEST GERMANY** (Region)					
*Bremen: City State*	2.863	2.863	3.151	3.072	2.815
*Hamburg: City State*	4.045	4.969	4.993	4.778	4.149
Baden‐Württemberg	4.527	5.078	5.127	4.908	4.396
Bayern [Bavaria]	4.734	5.228	5.324	5.114	4.570
Hessen [Hesse]	4.469	4.714	4.636	4.220	3.350
Niedersachsen [Lower Saxony]	4.511	4.822	4.631	4.424	3.882
Nordrhein‐Westfalen [North Rhine‐Westphalia]	3.975	4.177	3.822	3.458	2.946
Rheinland‐Pfalz [Rhineland‐ Palatinate]	3.040	3.393	3.318	3.144	2.657
Saarland	5.387	6.163	6.351	6.171	5.693
Schleswig‐Holstein	4.159	4.699	4.759	4.423	3.848
**Average (without Bremen & Hamburg)**	4.473	4.928	4.893	4.621	4.034
**Average (with Bremen & Hamburg)**	4.290	4.727	4.724	4.479	3.924

*Note*. East Germany states (estimated LTBI 22.5%^9^, consistently reported lower i‐CFR as compared to the West Germany States (estimated LTBI 9.2% LTBI.^7^ Eastern Germany with higher trained immunity correlates consistently had 20–30% lower CFR as compared to Western Germany states. The inclusion of Berlin in the East Germany region, and of Hamburg and Bermen in the West Germany region decreased the closing trend of the i‐CFR with the passage of time (compare covariation of red and green trend lines with orange and light green in Figure 4C) that could be reflective of more LTBI positives in Berlin as compared to Hamburg and Bremen.

## 4 CONCLUSIONS

In conclusion, we believe the incidences, mortality, and i‐CFR of COVID‐19 would negatively correlate with the trained immunity of populations that have comparable underlying confounders, not the BCG coverage *per se* till the populations remained naïve to SARS‐CoV‐2 infections and populations responded more equally. To decisively arrive at dependable conclusions about the potential protective benefit of BCG in COVID‐19, the ongoing or planned randomized 28 controlled trials (Supporting information Figure S2, Supplementary Tables 1–4) should consciously consider including measures of TIC^10^, ^24^ as— (a) all individuals immunized do not respond equally (up to 10–15% could be non‐responders), (b) small study groups of higher background trained immunity could fail to indicate any protective effect. Additionally, the inclusion of individuals who might have been exposed previously/recently to SARS‐CoV‐2 (asymptomatic or symptomatic) would tend to skew the trial outcomes toward displaying non‐protection. In concurrence with our assertions, the TST positivity has been recently observed to be associated with three times lower SARS‐CoV‐2 infections and protection from severe COVID‐19.^25^ Currently, under development COVID‐19 vaccines still have a long way to go and be available in sufficient supply to cover the whole global population at the same time to confer the much‐needed and touted ‘herd immunity’ whereas BCG is readily available which can be scaled up at a lower cost to provide the needed respite to vulnerable populations, especially in poor countries. Any potential protective effect displayed by BCG vaccination in the ongoing trials, especially in the aged and persons with comorbidities who are currently accounting for more than 90% of deaths, could help to provide hope in the current scenario. Nevertheless, study design improvements remain desired for increasing our confidence in the outcomes of the ongoing clinical trials evaluating the potency of BCG for COVID‐19 control.

## CONFLICT OF INTEREST

The authors declare no conflict of interest to disclose.

## Supporting information



Supporting information
