# Supplementary material for: Higher BCG‐induced trained immunity prevalence predicts protection from COVID‐19: Implications for ongoing BCG trials
Source: Clin Transl Discov. 2022 Jun 5;2(2):e60. doi: 10.1002/ctd2.60 (PMC9347530; doi:10.1002/ctd2.60)
Supplement: Supplementary file 1 — Supporting information [file CTD2-2-0-s001.pdf]

Higher BCG-induced trained immunity predicts protection from COVID-19

Running title: BCG-induced trained immunity and COVID-19

Samer Singh[1*], Dhiraj Kishore[2], Rakesh K. Singh[3*], Chandramani Pathak[4] and Kishu Ranjan[5*]

[1] Centre of Experimental Medicine & Surgery, Institute of Medical Sciences, Banaras Hindu University, Varanasi -221005, India

[2] Department of General Medicine, Institute of Medical Sciences, Banaras Hindu University, Varanasi – 221005, India.

[3] Department of Biochemistry, Institute of Science, Banaras Hindu University, Varanasi – 221005, India
[4] Amity Institute of Biotechnology, Amity University, Haryana, Gurgaon-122413, India
[5] Department of Pathology, School of Medicine, Yale University, New Haven, CT 06519, USA

***Correspondence:**
Samer Singh, Centre of Experimental Medicine & Surgery, Institute of Medical Sciences, Banaras Hindu University, Varanasi -221005, India. Email: samer.singh10@bhu.ac.in
Rakesh K. Singh, Department of Biochemistry, Institute of Science, Banaras Hindu University, Varanasi – 221005, India. Email: rakesh_bc@bhu.ac.in
Kishu Ranjan, Department of Pathology, School of Medicine, Yale University, New Haven, CT 06519, USA. Email: kishu.ranjan@yale.edu

Supplementary Figure 1. The notification rate of incidences and deaths from COVID-19 as per European CDC during the waves of SARS-CoV-2 infections. EU/EEA and the UK: 14-day COVID-19 case and death notification rates. Regional totals as of 11 Oct 2020: 4 065 835 cases (earliest 25 Jan, latest 11 Oct 2020), 195 235 deaths (15 Feb, 11 Oct 2020).

Supplementary Figure 2: Global BCG vaccination studies as prophylaxis for COVID-19. Africa (number of studies 4), Europe (number of studies 11), Canada (number of studies 1), Mexico (number of studies 1), USA number of studies (4), Pacifica (number of studies 2), South America (number of studies 4) and India (number of studies 1). Source: https://ClinicalTrials.gov (accessed on 03/12/2022)

Supplementary Figure 1

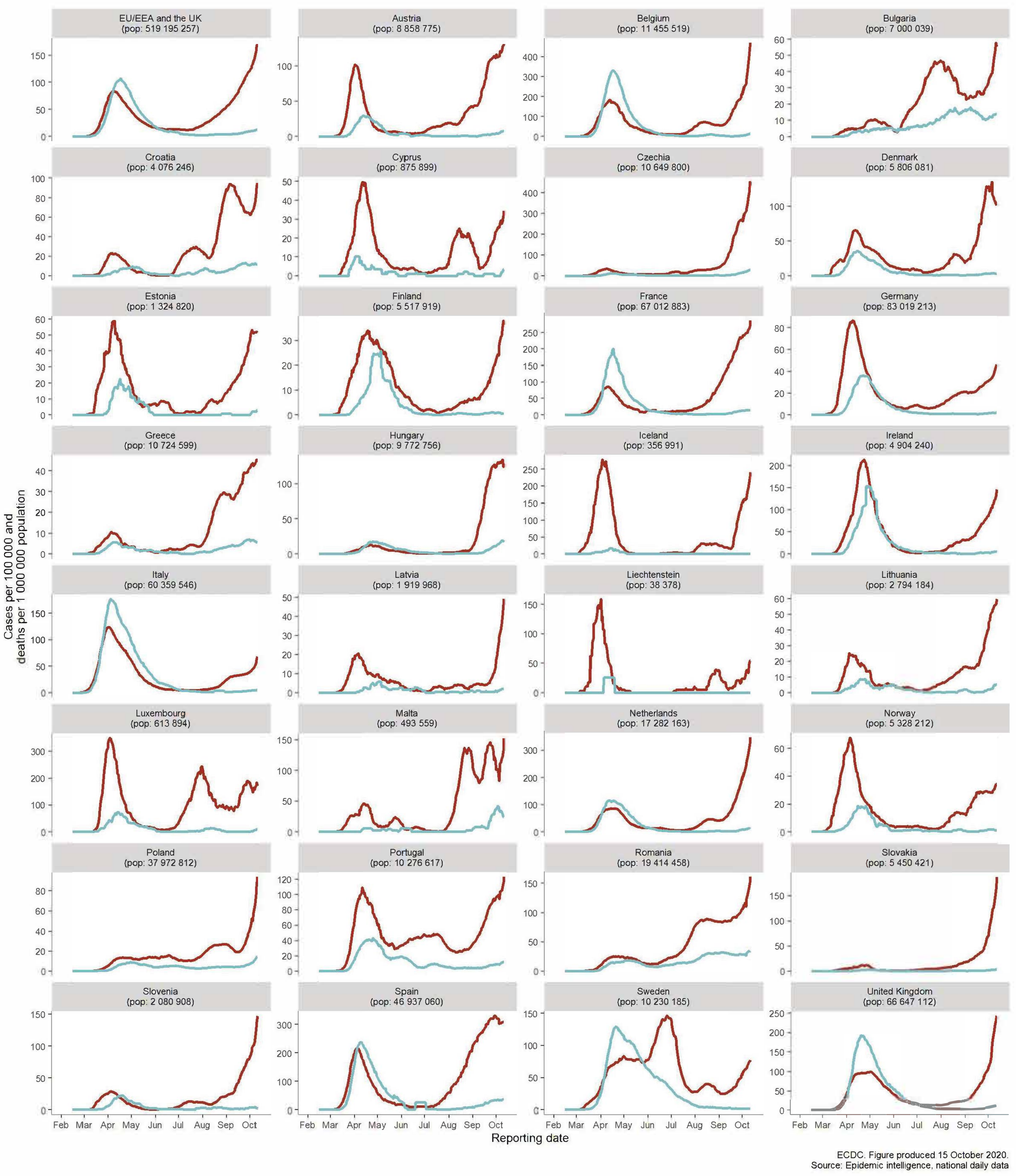

Supplementary Figure 2

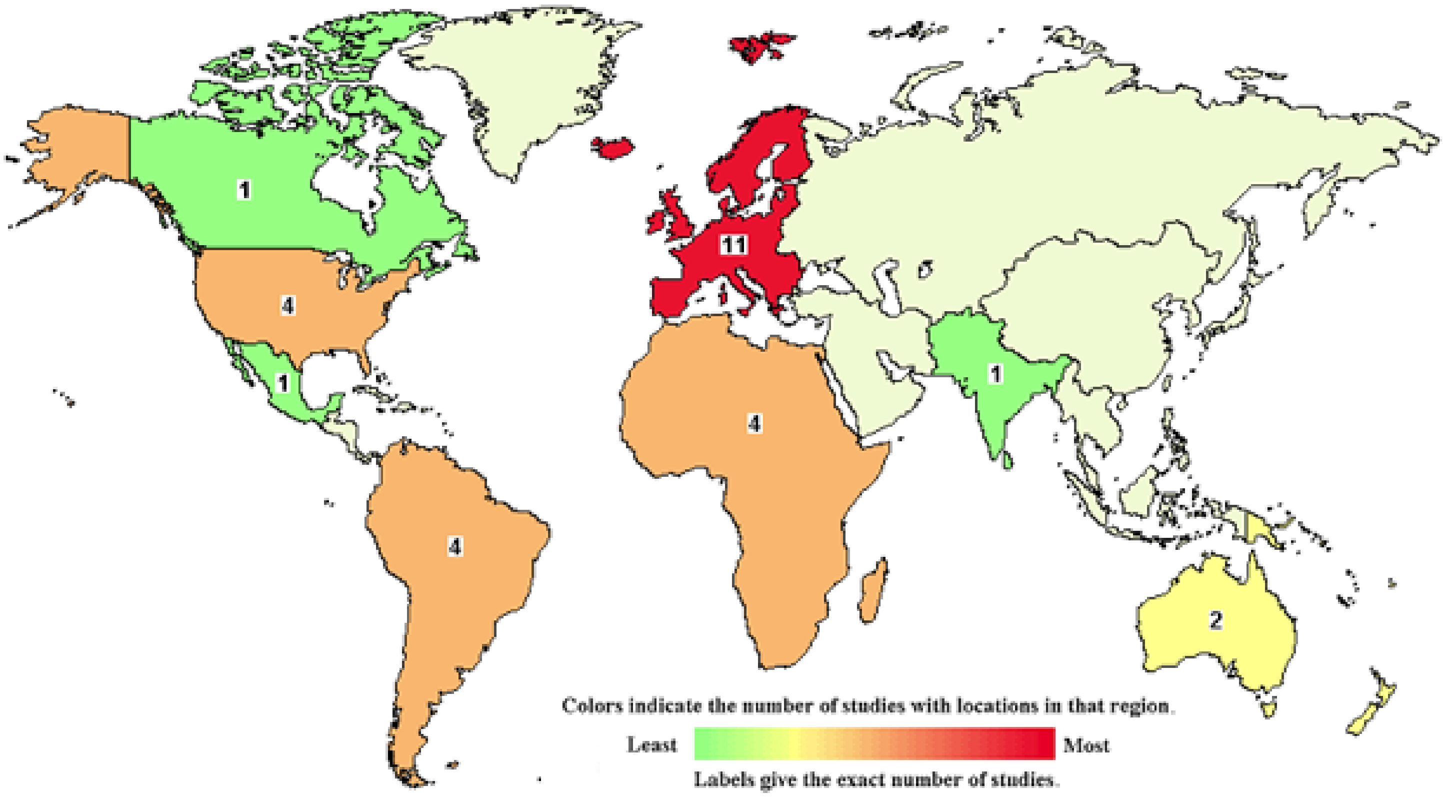

Supplementary Table-1: Clinical Trials on BCG vaccine in COVID-19 prevention (Completed)

NCT Number	Title	Phases	Enrollment	Start/End (MM-YY)	Locations
NCT04414267	Bacillus Calmette-guérin Vaccination to Prevent COVID-19.	Phase 4	301	May-20/ May-21	Greece
NCT04373291	Using BCG Vaccine to Protect Health Care Workers in the COVID-19 Pandemic.	Phase 3	1293	May-20/ Oct-21	Denmark
NCT04435379	Study to Assess VPM1002 in Reducing Hospital Admissions and/or Severe Respiratory Infectious Diseases in Elderly in COVID-19 Pandemic.	Phase 3	2038	Jun-20/ Oct-21	Germany
NCT04379336	BCG Vaccination for Healthcare Workers in COVID-19 Pandemic.	Phase 3	1000	May-20/ Jan-22	South Africa

NCT; National Clinical Trial, Jan; January, Jun; June, Oct; October, MM; Month, YY; Year.

Supplementary Table- 2: Clinical Trials on BCG vaccine in COVID-19 prevention (Recruiting)

NCT Number	Title	Phases	Enrollment	Start/End (MM-YY)	Locations
NCT04659941	Use of BCG Vaccine as a Preventive Measure for COVID-19 in Health Care Workers.	Phase 2	1000	Oct-20/ Oct-22	Brazil
NCT04369794	COVID-19: BCG As Therapeutic Vaccine, Transmission Limitation, and Immunoglobulin Enhancement.	Phase 4	1000	Aug-20/ Aug-23	Brazil
NCT04648800	Clinical Trial Evaluating the Effect of BCG Vaccination on the Incidence and Severity of SARS-CoV-2 Infections Among Healthcare Professionals During the COVID-19 Pandemic in Poland.	Phase 3	1000	Jul-20/ Apr-21	Poland
NCT04537663	Prevention of Respiratory Tract Infection And Covid-19 Through BCG Vaccination In Vulnerable Older Adults.	Phase 4	5200	Aug-20/ Jul-21	Netherlands
NCT04384549	Efficacy of BCG Vaccination in the Prevention of COVID19 Via the Strengthening of Innate Immunity in Health Care Workers.	Phase 3	1120	May-20/ Feb-21	France
NCT04542330	Using BCG to Protect Senior Citizens During the COVID-19 Pandemic	Phase 3	1900	Sep-20/ Mar-22	Denmark

NCT; National Clinical Trial, Feb; February, Mar, March, Apr; April, Jul; July, Aug; August, Sep; September, Oct; October, MM; Month, YY; Year.

Supplementary Table- 3: Clinical Trials on BCG vaccine in COVID-19 prevention (Active, not recruiting)

NCT Number	Title	Phases	Enrollment	Start/End (MM-YY)	Locations

NCT04328441	Reducing Health Care Workers Absenteeism in Covid-19 Pandemic Through BCG Vaccine.	Phase 3	1500	Mar-20/ May-21	Netherlands
NCT02403505	Early Phase Clinical Trial About Therapeutic Biological Product Mix for Treating COVID-19.	Early Phase 1	20	Dec-21/ Aug-22	USA
NCT04417335	Reducing COVID-19 Related Hospital Admission in Elderly by BCG Vaccination.	Phase 4	2014	Apr-20/ May-21	Netherlands
NCT04641858	BCG to Reduce Absenteeism Among Health Care Workers During the COVID-19 Pandemic.	Phase 4	668	Dec-20/ Aug-22	Mozambique
NCT04461379	Prevention, Efficacy and Safety of BCG Vaccine in COVID-19 Among Healthcare Workers.	Phase 3	908	Jul-20/ Jan-21	Mexico
NCT04327206	BCG Vaccination to Protect Healthcare Workers Against COVID-19.	Phase 3	10078	Mar-20/ Mar-22	Australia, Brazil, Netherland, Spain, UK
NCT05168709	Investigating COVID-19 Vaccine Immunity in Children in the Melbourne Infant Study of BCG for Allergy and Infection Reduction.	Phase 4	51	Jan-22/ May-22	Australia
NCT04348370	BCG Vaccine for Health Care Workers as Defense Against COVID 19.	Phase 4	1800	Apr-20/ May-22	USA
NCT04439045	Efficacy and Safety of VPM1002 in Reducing SARS-CoV-2 (COVID-19) Infection Rate and Severity.	Phase 3	122	Jun-20/ Dec-21	Canada
NCT04387409	Study to Assess VPM1002 in Reducing Healthcare Professionals' Absenteeism in COVID-19 Pandemic.	Phase 3	59	May-20/ May-21	Germany
NCT04445428	OPV as Potential Protection Against COVID-19.	Phase 4	3400	Jul-20/ Dec-21	Guinea-Bissau
NCT02081326	Repeat BCG Vaccinations for the Treatment of Established Type 1 Diabetes.	Phase 2	150	Jun-15/ Jul-27	USA
NCT04475302	BCG Vaccine in Reducing Morbidity and Mortality in Elderly Individuals in COVID-19 Hotspots.	Phase 3	2175	Jul-20/ Dec-21	India
NCT03348670	Discovery Stage COVID-19 Antigen Presentation Therapeutic Biologics Mix to Treat COVID-19 Virus Infection.	Early Phase 1	20	Oct-21/ Mar-22	USA

NCT; National Clinical Trial, Jan; January, Mar, March, Apr; April, Jun; June, Jul; July, Aug; August, Oct; October, Dec; December, MM; Month, YY; Year.

Supplementary Table- 4: Clinical Trials on BCG vaccine in COVID-19 prevention (Not yet recruiting/ Withdrawn)

NCT Number	Title	Phases	Enrollment	Start/End (MM-YY)	Locations

NCT04350931	Application of BCG Vaccine for Immune-prophylaxis Among Egyptian Healthcare Workers During the Pandemic of COVID-19.	Phase 3	900	Apr-20/ Dec-20	Egypt
NCT04534803	BCG Against Covid-19 for Prevention and Amelioration of Severity Trial (BAC to the PAST).	Phase 3	0	Sep-21/ Jul-22	
NCT04362124	Performance Evaluation of BCG Vaccination in Healthcare Personnel to Reduce the Severity of COVID-19 Infection.	Phase 3	0	Aug-20/ Nov-21	Colombia
NCT04632537	BCG Vaccination to Prevent COVID-19	Phase 3	0	Dec-20/ Mar-21	

NCT; National Clinical Trial, Mar, March, Apr; April, Jul; July, Aug; August, Sep; September, Nov; November, Dec; December, MM; Month, YY; Year.
